# MicroRNAs as biomarkers for major depression: a role for let-7b and let-7c

**DOI:** 10.1038/tp.2016.131

**Published:** 2016-08-02

**Authors:** A Gururajan, M E Naughton, K A Scott, R M O'Connor, G Moloney, G Clarke, J Dowling, A Walsh, F Ismail, G Shorten, L Scott, D M McLoughlin, J F Cryan, T G Dinan

**Affiliations:** 1 Department of Anatomy and Neuroscience, University College Cork, Cork, Ireland; 2 Department of Psychiatry and Neurobehavioural Science, University College Cork, Cork, Ireland; 3 St Patrick's University Hospital, Dublin, Ireland; 4 APC Microbiome Institute, University College Cork, Cork, Ireland; 5 Department of Anaesthesia and Intensive Care Medicine, University College Cork, Cork, Ireland; 6 Trinity College Institute of Neuroscience, Trinity College Dublin, Dublin 2, Ireland

## Abstract

There is a growing emphasis in the field of psychiatry on the need to identify candidate biomarkers to aid in diagnosis and clinical management of depression, particularly with respect to predicting response to specific therapeutic strategies. MicroRNAs are small nucleotide sequences with the ability to regulate gene expression at the transcriptomic level and emerging evidence from a range of studies has highlighted their biomarker potential. Here we compared healthy controls (*n*=20) with patients diagnosed with major depression (*n*=40) and who were treatment-resistant to identify peripheral microRNA biomarkers, which could be used for diagnosis and to predict response to electroconvulsive therapy (ECT) and ketamine (KET) infusions, treatments that have previously shown to be effective in treatment-resistant depression (TRD). At baseline and after treatment, blood samples were taken and symptom severity scores rated using the Hamilton Depression Rating Scale (HDRS). Samples were analyzed for microRNA expression using microarray and validated using quantitative PCR. As expected, both treatments reduced HDRS scores. Compared with controls, the baseline expression of the microRNA let-7b was less by ~40% in TRD patients compared with controls. The baseline expression of let-7c was also lower by ~50% in TRD patients who received ECT. Bioinformatic analysis revealed that let-7b and let-7c regulates the expression of 27 genes in the PI3k-Akt-mTOR signaling pathway, which has previously been reported to be dysfunctional in depression. The expression of miR-16, miR-182, miR-451 and miR-223 were similar to that in controls. Baseline microRNA expression could not predict treatment response and microRNAs were unaffected by treatment. Taken together, we have identified let-7b and let-7c as candidate biomarkers of major depression.

## Introduction

Depression is the most prevalent psychiatric disorder and current projections indicate that it will be the leading cause of disability by the year 2030.^1^ Subjective diagnostic schemes such as DSM-IV, ICD-10 are constrained in their ability to accurately diagnose depression^2^ and its subtypes such as treatment-resistant depression (TRD), which affects a significant proportion of patients.^3, 4^ As such, the potential benefits of using biomarkers to improve diagnostic precision and refine therapeutic strategies are significant.^5^ In this regard, emerging evidence from a range of clinical studies has reported the potential utility of microRNAs as biomarkers for a range of psychiatric disorders including depression.^6, 7^

MicroRNAs are small non-coding nucleotide sequences (18–24 nt), which regulate the expression of ~60% of the mammalian genome.^8^ Each microRNA can alter the translation of multiple messenger RNAs (mRNA) into proteins and each mRNA is the target of multiple microRNAs. This has led microRNAs to being known as ‘master-regulators' of cellular processes.^9^ The existence of microRNAs in bodily fluids such as blood and saliva^10^ has provided the impetus to evaluate their potential as biomarkers of illnesses and to predict response to different therapeutic strategies. In the context of depression, several microRNAs have shown biomarker potential including miR-16,^11^ miR-182,^12^ miR-223 and miR-451.^13^ There is also emerging evidence that basal microRNA expression can predict therapeutic response to antidepressants,^14^ consistent with their putative role as mediators of antidepressant effects.^15^ Moreover, several preclinical studies have shown that microRNAs mediate the antidepressant effects of electroconvulsive therapy (ECT, electroconvulsive stimulation in rodents)^16^ and the NMDA-receptor antagonist, ketamine (KET),^16, 17, 18^ therapeutic strategies, which have previously shown efficacy in TRD patients.^19, 20, 21^

Against this background, the aims of our study were to identify microRNA biomarkers that could be used for diagnosis of major depression as well as to predict response to treatments with ECT or KET.

## Materials and methods

**Figure 1:**
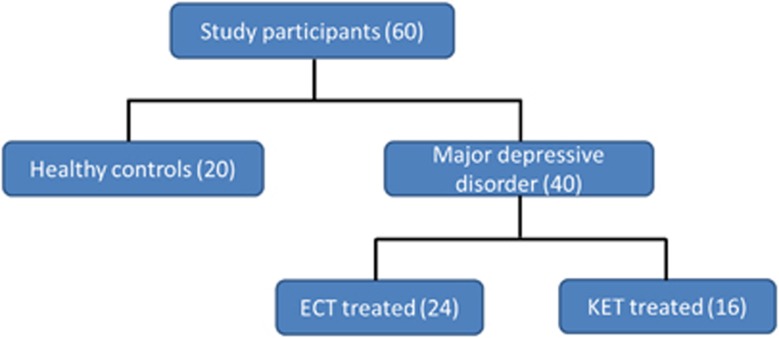
Breakdown and number of study participants according to treatment received. ECT, electroconvulsive therapy; KET, ketamine.

The study design (Figure 1), recruitment process and treatment procedures have been described previously.^21^ Briefly, patients who received KET treatment (*n*=16) were recruited from a mental health service in Cork, Ireland. Patients who received ECT (*n*=24) were inpatients at St Patrick's University Hospital. All patients had a diagnosis of major depressive disorder and had failed at least two adequate trials of antidepressant medication. Patients in the ECT group were older than healthy controls and patients in the KET group (Table 1). The majority of patients were receiving antidepressant therapy. Controls were recruited from Cork University Hospital staff and were screened for a personal or family history (1st degree relative) of a mental disorder and excluded if positive. Informed consent was obtained from all participants. There was no difference in gender profile between groups.

The ketamine component of the study was approved by the Clinical Research Ethics Committee of the Cork Teaching Hospitals (EMC (3nn) 08/11/11) and the Irish Medicines Board (IMB: EudraCT number: 2011-003654-40). The ECT component of the study was approved by the Ethics Committee of St Patrick's University Hospital (Protocol No. 21/12).

Patients received either KET intravenously (0.5 mg kg^−1^) once a week for up to three sessions, or bi-weekly brief-pulse bitemporal ECT based on published protocols. Blood samples were collected from all study participants at baseline between 0800 and 1100 hours on the morning of the first visit, prior to the first treatment session. Blood samples were obtained from KET-treated patients for microRNA analysis 24 h after the first infusion. For patients who received ECT treatment, samples were collected 4–7 days after the final session. For all patients, depression was assessed using the Hamilton Depression Rating Scale (HDRS) at the same time points at which blood was collected. Baseline HDRS scores were similar between the two groups of patients (Table 1). For patients who received KET treatment, additional HDRS scores were obtained 1 week after the first infusion. Treatment response was classified as at least a 50% reduction in HDRS scores relative to baseline. All blood samples were collected in PAXgene blood RNA tubes (PreAnalytix, Hombrechtikon, Switzerland) and stored at −80 °C until processing.

### Blood microRNA extraction

Total mRNA was isolated from blood samples using PAXgene Blood miRNA Kit (PreAnalytix). Samples were left at room temperature overnight prior to RNA extraction. Isolation was performed according to the protocol provided with the kit. Samples were eluted in 80 μl of buffer solution and stored in aliquots at −80 °C. Total RNA yield and quality were verified using the Nanodrop2000 spectrophotometer (ThermoScientific, Waltham, MA, USA) and RNA Integrity Number (RIN value) was assessed using the Agilent 2100 Bioanalyzer (Agilent, Santa Clara, CA, USA). Only samples with a RIN value ⩾7 were used for microarray analysis.

### Microarray analysis

MicroRNA Array analysis was completed by Exiqon Services (Exiqon, Vedbæk, Denmark). The quality of the total RNA was verified by an Agilent 2100 Bioanalyzer profile. Four hundred nanograms of total RNA from both sample and reference was labeled with Hy3TM and Hy5TM fluorescent label, respectively, using the miRCURY LNA microRNA Hi-Power Labeling Kit, Hy3TM/Hy5TM (Exiqon) following the procedure described by the manufacturer. The quantified signals were background corrected (Normexp with offset value 10) and normalized using the global Lowess (LOcally WEighted Scatterplot Smoothing) regression algorithm. Array data have been deposited in the NCBI's Gene Expression Omnibus (GSE81152).

### MicroRNA quantification

Quantitative real-time PCR (qRT-PCR) was carried out on available samples using probes (6 carboxy fluorescein-FAM) designed by Applied Biosystems (Carlsbad, CA, USA): miR-16 (assay ID:000391), miR-182 (assay ID:002334), miR-451 (assay ID:464419_mat), miR-223 (assay ID:002098), let-7b (assay ID:002619) and let-7c (assay ID:000379). RNA was reverse-transcribed to complementary DNA using hairpin primers specific to each microRNA gene of interest on Applied Biosystem's GeneAmp PCR System 9700. qRT-PCR was carried out on the StepOnePlus PCR machine (Applied Biosystems). Samples were heated to 95 °C for 10 min, and then subjected to 40 cycles of amplification by melting at 95 °C and annealing at 60 °C for 1 min. Experimental samples were run in technical replicates with 1.33 μl complementary DNA per reaction. To check for amplicon contamination, each run also contained template free controls for each probe used.

### Bioinformatic analysis

Bioinformatic analysis was performed using MicroT-CDS (v5.0) and the DIANA miRPath server (v3.0). MicroT-CDS is a database of over 11 million predicted *in silico* interactions between microRNAs and the 3′-UTR of their target mRNAs across a range of different species.^22^ miRPath uses a statistical algorithm which combines the Fisher's exact test, EASE Scores and false discovery rates to assign miRNA targets to biological pathways provided by the Kyoto Encyclopaedia of Genes and Genomes.^23, 24^ All identified pathways are arranged according to enrichment statistical scores (*P*-values) in addition to the number and names of miRNA target genes implicated in each Kyoto Encyclopaedia of Genes and Genomes pathway. For multiple microRNAs, the miRPath server calculates significance levels between each microRNA and every pathway. A merged *P*-value is then calculated and signifies if a particular pathway is targeted by at least one or more of the selected microRNAs. Pathways with a *P*-value <0.05 were considered. Gene ontology analysis of identified miRNA targets was performed with a focus on molecular function^25^ and interactions between targets using string analysis were examined.^26^

### Statistical analysis

Relationships between the various categorical variables were evaluated using the *χ*^2^-test. Age and HDRS scores (baseline and post treatment) between groups were analyzed using Student's *t*-test for parametric data and Mann–Whitney tests for the non-parametric data.

For microarray data, statistical analyses were performed using a two-way analysis of variance. *P*-values were corrected for multiple testing by the Benjamini and Hochberg adjustment method. Genes found to be significant by the one-way analysis of variance test have been subjected to the Tukey's ‘Honest Significant Difference' test to determine which groups contribute most to the significant difference. All calculations have been done in the software R/bioconductor using the limma package. To enable quick visual identification of those microRNAs that display large-magnitude changes that are also statistically significant, the expression data have been plotted in a Volcano plot (−log_10_(*P*-value) versus log_2_(fold-change)). We only quantified with qRT-PCR the expression of microRNAs that were were found to differentially expressed between groups (*P*<0.05).

PCR data were analyzed using the 2^−ΔCt^ method and outliers were defined by Grubb's method as previously described.^27^ The microRNA miR-25, which was stably expressed in all samples, was used as the endogenous control. Differences in microRNA expression between groups were determined using Student's *t*-test for parametric data and Mann–Whitney's test for the non-parametric data. Effects of treatments were determined using Student's *t*-test for parametric data and Mann–Whitney tests for the non-parametric data. To explore the associations between baseline microRNA expression and other clinical factors (for example, age and gender), Pearson's test was used for normally distributed data and Spearman's test was used for the non-normally distributed data. All statistics were calculated using SPSS-18 (IBM, Chicago, IL, USA). A *P*-value of 0.05 was selected as the threshold of statistical significance.

## Results

### Clinical effects of ketamine and ECT

Both ECT and KET treatments reduced the HDRS in the majority of patients (*P*<0.001; Table 1). There was no difference in post-treatment HDRS scores between the groups of TRD patients. There were no correlations between age, gender or medication profile and the ECT/KET-induced reduction in HDRS scores. Overall, there were more responders than non-responders. Five out of 11 patients demonstrated a sustained reduction 1 week after the first ketamine session.

### Microarray analysis

**Figure 2:**
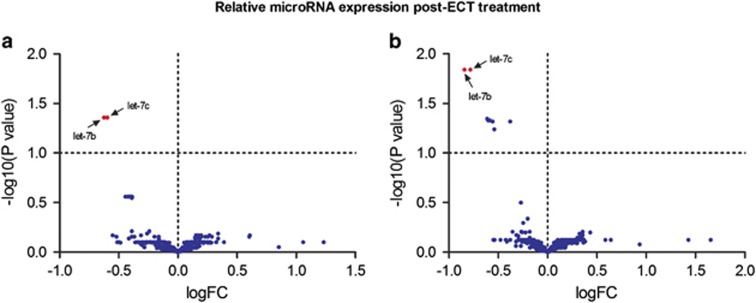
Microarray volcano plot showing differential expression of let-7b and let-7c (red) in (**a)** responders and (**b**) non-responders to ECT compared with healthy controls. ECT, electroconvulsive therapy.

A total of 502 out of 2087 possible microRNAs were detected in the blood samples. After controlling for false discovery rates with the Benjamini–Hochberg correction (see Materials and Methods), the only significant finding was a reduction in the expression of let-7b and let-7c in all TRD patients who received ECT (responders and non-responders; Figure 2, Supplementary Table 1). No microRNAs identified at baseline in TRD patients were predictive of response to ECT or KET and there were no other microRNAs that were affected by treatments.

### qRT-PCR analysis

**Figure 3:**
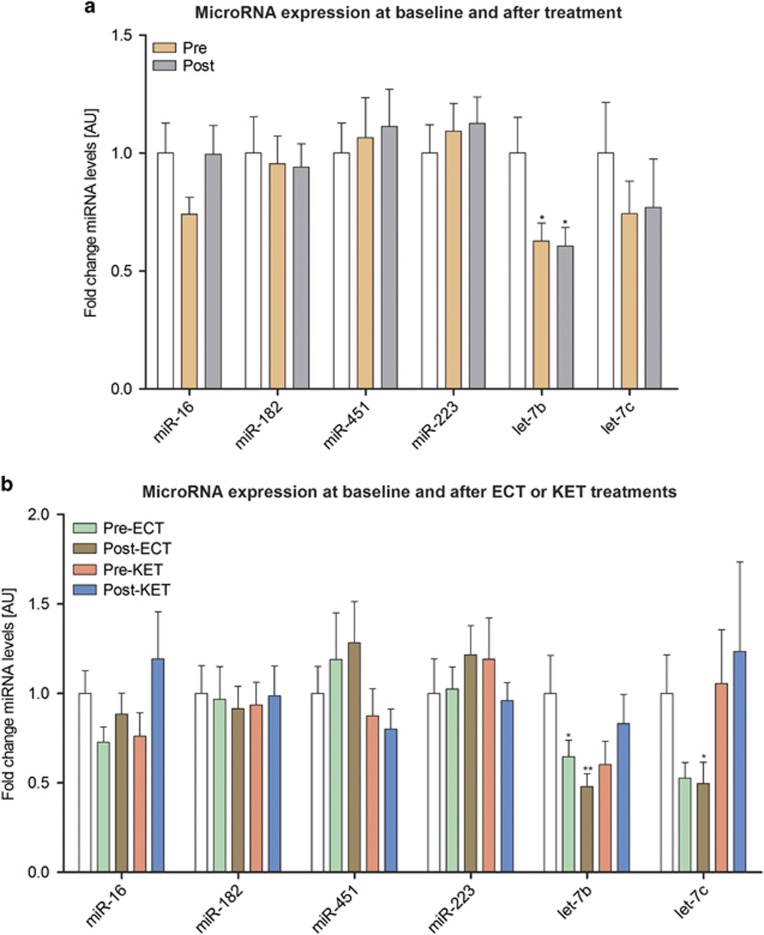
qRT-PCR data of microRNA expression (mean±s.e.m.) in controls (white column) and in (**a**) all patients at baseline (Pre) and after treatment (Post) and (**b**) according to type of treatment received (ECT or KET). *P<0.05, **P<0.01 compared with controls. ECT, electroconvulsive therapy; KET, ketamine; qRT-PCR, quantitative real-time PCR.

On the basis of microarray analysis, we first quantified baseline differences in the expression of let-7b and let-7c in all TRD patients compared with controls. The expression of let-7b was less in TRD patients compared with that in controls; there was no difference in let-7c expression between patients and controls (Figure 3a, Supplementary Table 2).

When patient groups were split according to the treatments they subsequently received, baseline expression let-7b was lower in patients who progressed to receive ECT treatment (Figure 3b, Supplementary Table 3). In patients who received KET treatment, there was a trend towards a lower baseline expression of let-7b. There was no difference in baseline let-7b expression between these two patient groups. Given that the age of patients in the ECT group were older than controls and patients in the KET group, we assessed whether there was a correlation between age and baseline expression of let-7b, but this was found to be not statistically significant. Patients in the ECT group were also receiving more medication, but there was no significant correlation between medication profile and let-7b expression. Compared with controls the baseline expression of let-7c tended to be lower in patients in the ECT group. In addition to let-7b and let-7c we also quantified the expression of miR-16, miR-182, miR-451 and miR-223, all of which have been implicated in depression^7^ and found that their baseline expression was similar to those of controls (Figures 3a and b).

Overall, there were no microRNAs affected by KET or ECT treatments. However, there was a trend toward higher post-treatment expression of let-7b in patients who had received KET than ECT. This increase in let-7b expression was subsumed into the post-ECT group, which could explain the group significance seen in Figure 3a.

**Figure 4:**
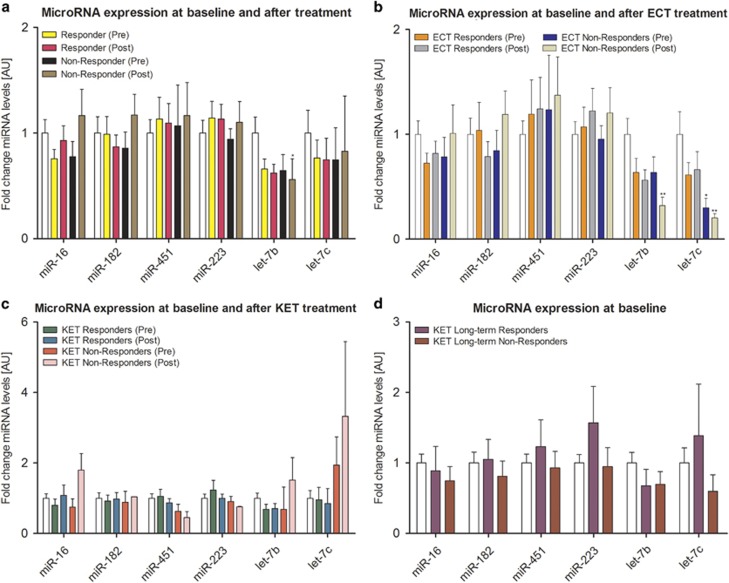
qRT-PCR data of microRNA expression (mean±s.e.m.) in controls (white column) and in all (**a**) patients, (**b**) ECT patients and (**c**) KET patients at baseline and after treatment according to response. (**d**) Baseline microRNA expression in long-term responders and non-responders to KET treatment. *P<0.05, **P<0.01 compared with controls. ECT, electroconvulsive therapy; KET, ketamine; qRT-PCR, quantitative real-time PCR.

We next determined whether baseline microRNA expression could predict response to treatment in TRD patients. Relative to controls, there was a trend towards lower expression of let-7b in responders but overall, the baseline expression of both let-7b and the other microRNAs did not differ significantly between responders and non-responders. In both responders and non-responders, there was no effect of treatment, but post-treatment expression of let-7b was reduced compared with controls (Figure 4a, Supplementary Table 4).

When we stratified the analysis according to the types of treatment, in patients who received ECT, baseline expression of let-7c was low in non-responders relative to controls (Figure 4b, Supplementary Table 5). However, the overall baseline expression of all microRNAs was similar in responders and non-responders. In responders, ECT treatment had no effect on microRNA expression. In non-responders, post-treatment expression of let-7b and let-7c was less compared with that of controls, but pre- and post-treatment levels were similar.

For patients who received KET treatment, there were no differences in the baseline expression of all microRNAs between responders and non-responders (Figure 4c, Supplementary Table 6). Similarly, there was no effect of treatment on microRNA expression in responders or in non-responders. Last, we examined whether baseline microRNA expression could predict response to KET at 1 week and found there were no differences between those who exhibited a sustained reponse and non-responders at this later time point (Figure 4d, Supplementary Table 7).

Taken together, these results would suggest that microRNAs let-7b and let-7c are potential biomarkers of TRD. Baseline expression of let-7b, let-7c or any other microRNAs in TRD did not predict treatment response. Interestingly, both the direction and the magnitude of the changes in the microarray were significantly different from qRT-PCR analyses. In particular, whereas microarray results showed a significant reduction in let-7b and let-7c expression following ECT treatment, qRT-PCR analysis did not detect this difference. We have addressed this discrepancy in detail in our discussion.

### Bioinformatics analysis of let-7b and let-7c gene targets and pathways

**Figure 5:**
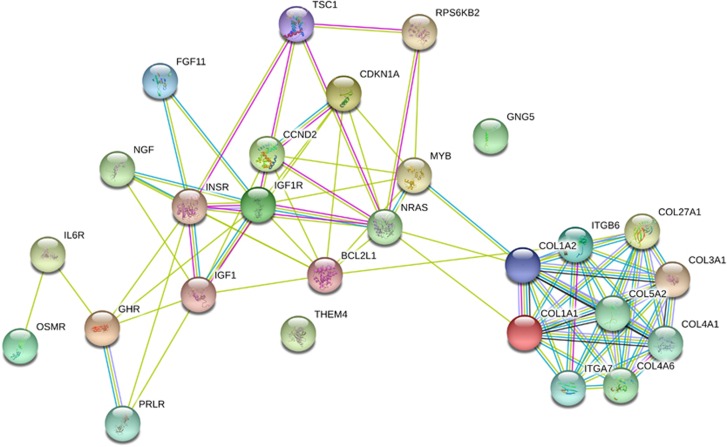
String analysis showing interactions between identified genes under the regulation of let-7b and let-7c. Darker lines indicate stronger network connections between genes.

Bioinformatics analysis revealed that there are 1343 genes targeted by let-7b (682) and let-7c (661). Pathway union analysis revealed a significant over-representation of 27 genes (Table 2) in the PI3k-Akt signaling pathway. Gene ontology analysis revealed that the 12 out of the 27 genes (Table 3) were involved in receptor activity and protein binding. String analysis showed that genes involved in structural molecular activity and composition of the extracellular matrix, such as collagen and integrin, clustered together in terms of their predicted interactions. A separate and larger cluster included genes for receptors implicated in other molecular functions including those for hormones and growth factors such as the growth hormone, insulin and insulin-like growth factor (Figure 5).

## Discussion

In this study, we sought to identify microRNAs that were differentially expressed in patients with major depression and TRD compared with controls and which were predictive of response to either ECT or KET treatments. We observed that baseline expression of let-7b was significantly lower in all patients compared with healthy controls. When analysis was split according to the type of treatments received, let-7b expression was comparable between these two groups. This was significant in the ECT group and displayed a strong trend toward significance in the KET group. The baseline expression of let-7c was also lower in patients who subsequently received ECT compared with controls and was unaffected by this treatment. These findings suggest that let-7b and let-7c are candidate biomarkers of major depression. We also speculate as to whether they may also be biomarkers for TRD. However, our conclusions come with several limitations discussed below.

The let-7 family of microRNAs were the first to be discovered in humans with roles in neurogenesis and synapse formation.^28, 29^ One previous study has shown dysregulation in another member of the let-7 family, let-7p-5p, in patients with schizophrenia,^30^ but to our knowledge, there have been no investigations into whether this family of microRNAs is dysregulated in depression. However, they have been shown to be responsive to a variety of mood stabilizers and antidepressants.^31^ There are ~1343 predicted experimental targets of let-7b and let-7c, but pathway analysis revealed that there is a significant overexpression of 27 genes in the intracellular PI3K-Akt signaling pathway. Moreover, one of the downstream targets of activated Akt is the mTOR signaling pathway, which has been previously implicated in the pathophysiology of depression^32, 33^ and the rapid antidepressant effects of KET.^34^

Gene ontology analysis revealed that the 12 out of the 27 genes were involved in binding of growth factors such as insulin and growth hormone (GH). Evidence suggests that the insulin-like growth factor (IGF1) can promote the signaling effects of the brain-derived neurotrophic factor and also act synergistically with brain-derived neurotrophic factor to induce antidepressant-like effects.^35^ Both IGF1 and insulin can bind to IGF1 and insulin receptors (IGF1R and INSR) to activate downstream signaling cascades including the PI3k-Akt signaling pathway.^36, 37^ Dysregulation of GH release from the anterior pituitary is associated with atypical depression.^38^ In depressed patients, secretion is reportedly abnormal, but this appears to depend on the age of the patients; studies show significantly reduced secretion of GH in depressed children and adolescents,^39^ but no change in adults.^40, 41^ The activation of the PI3K-Akt signaling pathway by GH is indirect via JAK2 tyrosine kinase phosphorylation which in turn phosphorylates insulin-like receptor substrates.^42^ String analysis showed that genes for several collagen factors clustered together. Collagen is a structural protein found in the basement membranes of blood vessels and given that the current study utilized blood samples for microRNA analysis, this finding is expected.

The ability to predict treatment response based on expression of biomarkers confers clinical advantage in terms of choosing the right therapeutic strategy for the right patient. We explored this possibility in our study, but identified no microRNAs at baseline that predicted response to ECT or KET treatments. To our knowledge, there has been only one study that studied microRNAs as biomarkers to predict antidepressant response. Lopez *et al.*^14^ showed that baseline expression of miR-1202 was lower in patients with depression who subsequently responded to an 8-week regimen of the selective serotonin reuptake inhibitor, citalopram. However, patients were drug-naive at study commencement. They were classified as responders and non-responders based on changes to HDRS scores following treatment, but it is unclear what their cutoff criteria were for response and non-response. Previous studies have used cutoffs ranging from 30 to 60% improvement in HDRS scores^43, 44^ to distinguish responders from non-responders. For our study, our classification was based on at least a 50% reduction in HDRS scores.^21^

Our results with miR-16 are consistent with recent work, which reported that expression of miR-16 in the blood was not different between controls and patients with major depressive disorder.^11^ The fact that peripheral miR-16 expression is unchanged relative to controls in major depression and TRD argues against its involvement in the pathophysiology. However, its expression was found to be reduced in the cerebrospinal fluid of patients with major depression.^11^ Overlaps in the transcriptome profiles between the periphery and the brain is reportedly high (81.9%),^45^ but this does not imply that alterations in the profile in one compartment will be mirrored in the other, either in the context of health or during periods of illness. Although its role as a diagnostic biomarker remains to be validated, previous work has shown that miR-16 is a molecular mediator of the antidepressant effect, particularly for selective serotonin reuptake inhibitors.^46^ Our results suggest that peripheral miR-16 levels do not reflect a potential role in the therapeutic mechanisms of ECT or KET treatment.

In contrast to previous studies, we observed no differences in expression of miR-182 and miR-223 between patients and controls.^12, 13^ Potential causes for the discrepancy include medication status at study commencement (drug-naive versus medicated), sampling source (for example, serum and plasma) and the fact that patients in our study were approximately 10–20 years older. Another important factor which has been discussed in other disease contexts, but not in the depression-microRNA literature is ethnicity.^47, 48, 49^ This study was conducted in Ireland, whereas the previous studies were conducted in China and Turkey.

We have previously shown that the maternal separation paradigm in rats can induce significant down-regulation of miR-451 expression in the hippocampus,^16^ a brain region that is consistently implicated in depression.^50, 51^ In contrast, a recent study has shown increased peripheral expression of miR-451 in patients with depression.^13^ We found no differential expression of miR-451 between patients and controls, but factors already described (for example, age and sampling source) could explain the discrepancy in clinical findings. The lack of consistency between clinical and preclinical studies suggests poor correlation between central and peripheral readouts of miR-451 expression. The expression of miR-451 was not altered following treatment with ketamine, a result which was consistent with our preclinical study, suggesting that it is unlikely to be involved in the therapeutic mechanism of this novel antidepressant.

The results of this study should be interpreted in the light of its limitations. The patients were on medication at the time of study commencement and throughout the study itself. This was owing to ethical considerations clinically but could have confounded the results. Furthermore, our patient group was heterogeneous with melancholic and non-melancholic patients included. Moreover, patients in the ECT group were older than all other study participants and were thus more likely to be on multiple medications for a variety of health conditions. They were also institutionalized. Also, extraction of microRNAs for patients in the KET group was only 24 h after the first infusion whereas for the ECT group it was several days after treatment. These differences could explain the divergence in baseline and post-treatment microRNA expression levels for let-7b and let-7c. Moreover, we did not include patients who were responsive to conventional antidepressants in our study to support our hypothesis that let-7b and let-7c were also potential biomarkers for TRD. In addition, some factors inherent to the ECT procedure may also hinder a clear interpretation of the studies, that is, electrode placement, stimulus intensity and frequency, and seizure duration (which all may have different effects on microRNAs), as well as the use of general anaesthesia. We also did not assess patient insulin, IGF or GH levels, which could have supported the findings of the bioinformatics analyses. However, our approach of using only an *in silico* methodology to draw conclusions about the involvement of microRNAs in pathological processes has precedent.^13, 52, 53^ Given the role of stress as a major risk factor for the development of psychopathologies, we did not determine whether the patients had early-life or current stress experiences. To our knowledge, there have been no microRNA studies in clinical depression that have taken this variable into account. Last, we stress that these findings are preliminary and needs replication.

It is also important to note that the PCR results were in contrast to what was observed from microarray analysis. Fidelity between the two techniques is an ongoing issue due to a range of factors including the sensitivity of microarray probes to differentiate between mature and precursor microRNA sequences.^54^ It is also possible that pathology and medication could have had effects on microRNAs, which could only be detected by more accurate PCR that is considered a ‘gold-standard'.^55^

Although it is clear that microRNAs function as a mechanism for post-transcriptional regulation, it has not been conclusively proven whether, under conditions of homeostasis or pathology, their presence in body fluids is simply a by-product of cell degradation or whether are they actively secreted into the body fluids to mediate intercellular gene regulation. Nevertheless, the correlation between circulating microRNAs and peripheral tissue microRNAs suggests that in human fluids they might serve as biomarkers for various diseases.^56^ However, in the context of depression few studies have supported such correlations between circulating and central readouts of microRNA expression.^14, 57^ Indeed, of the microRNAs that we analyzed in this study, a correlation between changes in peripheral blood and in brain tissue remains to be established. Thus, in the case of let-7b and let-7c future clinical studies could focus on post-mortem samples from patients who had suffered from major depression to determine whether this microRNA is a valid diagnostic biomarker. *In vivo* and *in vitro* models could also be used to further investigate the functional expression of these microRNAs.

In conclusion, we provide preliminary evidence that let-7b and let-7c are candidate diagnostic biomarkers of major depression. Future studies utilizing larger patient samples with more detailed medical histories and the extraction of both peripheral blood samples and cerebrospinal fluid samples will allow us to validate let-7b, let-7c and other potential microRNAs that could be used for diagnosis, predict response to various therapeutic strategies and provide novel insights into the neuromolecular pathophysiology of depression.

## Figures and Tables

**Table 1:** Characteristics of healthy controls and patients

	*Controls (*n=*20)*	*ECT (*n=*24)*^a^	*Ketamine (*n=*16)*^b^	P*-value*
Age	42.85±2.22	56.88±2.75	44.63±3.40	Control vs ECT: *P*<0.01 ECT vs KET: *P*<0.01
Gender	11M, 9F	9M, 15F	10M, 6F	NS
Baseline HDRS	—	22.42±0.90	21.38±0.75	NS
				
*Post-treatment HDRS*	*—*	*9.26±1.26*	*7.15±0.84*	*NS*
Responder		5.67±0.78 (15/23)	6.36±0.76 (11/13) 5.2±0.58 (5/11 long-term responders)	NS
Non-responder		16.00±1.49 (8/23)	11.00±4.00 (2/13)	NS
				
*Medication profile*^c^				
Selective serotonin reuptake inhibitor		3	5	
Selective noradrenaline reuptake inhibitor		8	4	
Serotonin agonist and reuptake inhibitor		1	—	
Tricyclic antidepressant		7	—	
Noradrenergic and specific serotonergic antidepressant		4	1	
Monoamine oxidase inhibitor		1	—	
Buproprion		1	2	
Other		7	4	

Abbreviations: ECT, electroconvulsive therapy; F, female; HDRS, Hamilton Depression Rating Scale; KET, ketamine; M, male; NS, not significant.

a One sample from a patient post-ECT session was unavailable.

b Three samples from patient post-KET infusion were unavailable.

c Eight patients in the ECT group were on more than one of antidepressants listed in this table.

**Table 2:** Genes in the PI3K-Akt pathway under the regulation of let-7b and let-7c

*Gene ID*	*Description*
COL1A1	Collagen, type I, alpha 1
GHR	Growth hormone receptor
PRLR	Prolactin receptor
ITGB6	Integrin, beta 6
MYB	V-myb avian myeloblastosis viral oncogene homolog
CCND2	Cyclin D2
CDKN1A	Cyclin-dependent kinase inhibitor 1A (p21, Cip1)
NGF	Nerve growth factor
ITGA7	Integrin, alpha 7
IGF1R	Insulin-like growth factor 1 receptor
OSMR	Oncostatin M receptor
THEM4	Thioesterase superfamily member 4
IL6R	Interleukin 6 receptor
COL1A2	Collagen, type I, alpha 2
TSC1	Tuberous sclerosis 1
COL3A1	Collagen, type III, alpha 1
INSR	Insulin receptor
BCL2L1	BCL2-like 1
GNG5	Guanine nucleotide binding protein (G protein), gamma 5
RPS6KB2	Ribosomal protein S6 kinase, 70kDa, polypeptide 2
COL4A1	Collagen, type IV, alpha 1
COL27A1	Collagen, type XXVII, alpha 1
COL4A6	Collagen, type IV, alpha 6
COL5A2	Collagen, type V, alpha 2
NRAS	Neuroblastoma RAS viral (v-ras) oncogene homolog
FGF11	Fibroblast growth factor 11
IGF1	Insulin-like growth factor 1

**Table 3:** Gene ontology analysis of molecular function of identified targets of let-7b and let-7c

*Molecular function*	*Frequency*	P*-value*
Receptor activity (COL5A2, OSMR, IL6R, PRLR, COL4A6, COL4A1, COL3A1,COL27A1, ITGB6, COL1A2, COL1A1, GHR)	12/1636	5.30e−5
Protein binding (FGF11, BCL2L1, NRAS, NGF, OSMR, IL6R, GNG5, PRLR, CCND2, CDKN1A, IGF1, GHR)	12/2819	1.53e−2
Extracellular matrix structural constituent (COL5A2, COL4A6, COL4A1, COL3A1, COL27A1, COL1A2, COL1A1)	7/76	1.22e−9
Transmembrane transporter activity (COL5A2, COL4A6, COL4A1, COL3A1, COL27A1, COL1A2, COL1A1)	7/1010	4.02e−2
Receptor binding (FGF11, NGF, OSMR, IL6R, PRLR, IGF1, GHR)	7/980	3.34e2
Structural molecular activity (COL5A2, COL4A6, COL4A1, COL3A1, COL27A1, COL1A2, COL1A1)	7/1034	4.64e−2
Cytokine activity (NGF, OSMR, IL6R, PRLR, GHR)	5/230	1.84e−3

Frequency is based on number of genes identified relative to total number of reference genes in humans for each molecular function.
