# Supplementary material for: MicroRNAs as biomarkers for major depression: a role for let-7b and let-7c
Source: Transl Psychiatry. 2016 Aug 2;6(8):e862–. doi: 10.1038/tp.2016.131 (PMC5022079; doi:10.1038/tp.2016.131)
Supplement: Supplementary Information [file tp2016131x1.pdf]

Table 1 - Microarray expression of let-7b and let-7c following ECT treatment

	Responder		Non-responder	
**Probe**	**let-7b**	**let-7c**	**let-7b**	**let-7c**
**Average Hy3**	11.435	10.104	11.435	10.104
**LogFC**	-0.625	-0.597	-0.843	-0.783
**p-value**	0.043	0.043	0.014	0.014

Average Hy3: array signal intensity, LogFC:log2(foldchange)

Table 2 - qRT-PCRT MicroRNA expression at baseline and after treatment

	Relative fold change ± SEM (p-value)	
	**Pre**	**Post**
**miR-16**	0.741 ± 0.072 (0.128)	0.995 ± 0.122 (0.620)
**miR-182**	0.955 ± 0.118 (0.509)	0.94 ± 0.099 (0.674)
**miR-451**	1.065 ± 0.170 (0.584)	1.113 ± 0.158 (0.964)
**miR-223**	1.093 ± 0.118 (0.838)	1.126 ± 0.112 (0.733)
**let-7b**	0.627 ± 0.076 **(0.027)**	0.605 ± 0.079 **(0.011)**
**let-7c**	0.743 ± 0.138 (0.173)	0.77 ± 0.205 (0.106)

Table 3 - qRT-PCR MicroRNA expression at baseline and after ECT or KET treatment

	Relative fold change ± SEM (p-value)			
	**Pre-ECT**	**Post-ECT**	**Pre-KET**	**Post-KET**
**miR-16**	0.728 ± 0.085 (0.145)	0.883 ± 0.119 (0.374)	0.761 ± 0.130 (0.249)	1.192 ± 0.263 (0.786)
**miR-182**	0.968 ± 0.182 (0.430)	0.915 ± 0.125 (0.548)	0.937 ± 0.126 (0.782)	0.986 ± 0.167 (0.969)
**miR-451**	1.189 ± 0.262 (0.764)	1.283 ± 0.230 (0.593)	0.875 ± 0.152 (0.400)	0.801 ± 0.112 (0.373)
**miR-223**	1.025 ± 0.121 (0.711)	1.216 ± 0.163 (0.481)	1.191 ± 0.231 (0.940)	0.961 ± 0.099 (0.745)
**let-7b**	0.644 ± 0.094 **(0.044)**	0.478 ± 0.073 **(0.003)**	0.602 ± 0.131 (0.065)	0.832 ± 0.162 (0.329)[#]
**let-7c**	0.526 ± 0.087 (0.078)	0.495 ± 0.119 **(0.028)**	1.054 ± 0.300 (0.511)	1.235 ± 0.499 (0.614)

[#] *Let-7b: Post-ECT vs Post-KET, p=0.061*

Table 4 - qRT-PCR MicroRNA expression at baseline and after treatment

	Relative fold change ± SEM (p-value)			
	**Responder (Pre)**	**Responder (Post)**	**Non-Responder (Pre)**	**Non-Responder (Post)**
**miR-16**	0.757 ± 0.088 (0.187)	0.929 ± 0.141 (0.441)	0.776 ± 0.143 (0.384)	1.166 ± 0.249 (0.800)
**miR-182**	0.988 ± 0.167 (0.540)	0.869 ± 0.112 (0.413)	0.856 ± 0.155 (0.414)	1.171 ± 0.195 (0.469)
**miR-451**	1.134 ± 0.204 (0.956)	1.094 ± 0.187 (0.847)	1.069 ± 0.384 (0.521)	1.166 ± 0.312 (1.000)
**miR-223**	1.142 ± 0.158 (0.643)	1.134 ± 0.137 (0.494)	0.939 ± 0.100 (0.773)	1.103 ± 0.195 (0.893)
**let-7b**	0.659 ± 0.094 (0.082)	0.623 ± 0.082 (0.165)	0.645 ± 0.150 (0.113)	0.560 ± 0.196 **(0.034)**
**let-7c**	0.763 ± 0.169 (0.302)	0.747 ± 0.205 (0.169)	0.746 ± 0.304 (0.201)	0.827 ± 0.524 (0.113)

Table 5 - qRT-PCR MicroRNA expression at baseline and after ECT treatment

	Relative fold change ± SEM (p-value)			
	**ECT Responder (Pre)**	**ECT Responder (Post)**	**ECT Non-Responder (Pre)**	**ECT Non-Responder (Post)**
**miR-16**	0.726 ± 0.094 (0.114)	0.817 ± 0.117 (0.277)	0.785 ± 0.185 (0.177)	1.008 ± 0.270 (0.664)
**miR-182**	1.038 ± 0.267 (0.524)	0.787 ± 0.143 (0.231)	0.844 ± 0.913 (0.395)	1.189 ± 0.224 (0.506)
**miR-451**	1.192 ± 0.325 (0.844)	1.241 ± 0.299 (0.794)	1.233 ± 0.520 (0.725)	1.137 ± 0.365 (0.405)
**miR-223**	1.071 ± 0.187 (0.795)	1.221 ± 0.215 (0.539)	0.951 ± 0.131 (0.791)	1.202 ± 0.240 (0.815)
**let-7b**	0.636 ± 0.132 (0.097)	0.562 ± 0.098 (0.123)	0.636 ± 0.147 (0.177)	0.319 ± 0.081 **(0.003)**
**let-7c**	0.612 ± 0.116 (0.310)	0.662 ± 0.172 (0.220)	0.298 ± 0.089 **(0.016)**	0.203 ± 0.040 **(0.003)**

Table 6 - qRT-PCR MicroRNA expression at baseline and after KET treatment

	Relative fold change ± SEM (p-value)			
	**KET Responder (Pre)**	**KET Responder (Post)**	**KET Non-Responder (Pre)**	**KET Non-Responder (Post)**
**miR-16**	0.804 ± 0.173 (0.181)	1.082 ± 0.293 (0.552)	0.753 ± 0.231 (0.429)	1.800 ± 0.470 (0.103)
**miR-182**	0.922 ± 0.169 (0.692)	0.981 ± 0.183 (0.967)	0.884 ± 0.317 (0.732)	1.043
**miR-451**	1.052 ± 0.200 (0.945)	0.872 ± 0.120 (0.694)	0.629 ± 0.202 (0.282)	0.446 ± 0.177 (0.118)
**miR-223**	1.231 ± 0.278 (0.606)	1.002 ± 0.115 (0.646)	0.908 ± 0.150 (0.900)	0.755 ± 0.013 (0.864)
**let-7b**	0.687 ± 0.138 (0.213)	0.707 ± 0.140 (0.235)	0.684 ± 0.636 (0.134)	1.159 ± 0.630 (0.365)
**let-7c**	0.957 ± 0.357 (0.459)	0.854 ± 0.421 (0.258)	1.939 ± 0.798 (0.184)	3.325 ± 2.118 (0.094)

Table 7 - qRT-PCR MicroRNA expression at baseline

	Relative fold change ± SEM (p-value)	
	**KET Long-term Responder**	**KET Long-term Non-Responders**
**miR-16**	0.888 ± 0.348 (0.399)	0.748 ± 0.200 (0.176)
**miR-182**	1.053 ± 0.282 (0.833)	0.813 ± 0.215 (0.445)
**miR-451**	1.233 ± 0.379 (0.508)	0.931 ± 0.234 (0.768)
**miR-223**	1.572 ± 0.513 (0.246)	0.947 ± 0.271 (0.796)
**let-7b**	0.676 ± 0.235 (0.298)	0.697 ± 0.182 (0.350)
**let-7c**	1.387 ± 0.731 (0.899)	0.598 ± 0.233 (0.296)
